# Usage Intensity of a Relapse Prevention Program and Its Relation to Symptom Severity in Remitted Patients With Anxiety and Depression: Pre-Post Study

**DOI:** 10.2196/25441

**Published:** 2022-03-16

**Authors:** Esther Krijnen-de Bruin, Anna DT Muntingh, Evelien M Bourguignon, Adriaan Hoogendoorn, Otto R Maarsingh, Anton JLM van Balkom, Neeltje M Batelaan, Annemieke van Straten, Berno van Meijel

**Affiliations:** 1 Department of Psychiatry Amsterdam Public Health Research Institute Amsterdam UMC Amsterdam Netherlands; 2 Department of Research and Innovation GGZ inGeest Specialized Mental Health Care Amsterdam Netherlands; 3 Department of Health, Sports and Welfare Research Group Mental Health Nursing Inholland University of Applied Sciences Amsterdam Netherlands; 4 Department of General Practice Amsterdam Public Health Research Institute Amsterdam UMC Amsterdam Netherlands; 5 Department of Clinical, Neuro and Developmental Psychology VU University Amsterdam Amsterdam Public Health Research Institute Amsterdam Netherlands; 6 Parnassia Academy Parnassia Psychiatric Institute The Hague Netherlands

**Keywords:** relapse prevention, anxiety disorder, depressive disorder, eHealth, primary care practice, usage intensity, self-management, mobile phone

## Abstract

**Background:**

Given that relapse is common in patients in remission from anxiety and depressive disorders, relapse prevention is needed in the maintenance phase. Although existing psychological relapse prevention interventions have proven to be effective, they are not explicitly based on patients’ preferences. Hence, we developed a blended relapse prevention program based on patients’ preferences, which was delivered in primary care practices by mental health professionals (MHPs). This program comprises contact with MHPs, completion of core and optional online modules (including a relapse prevention plan), and keeping a mood and anxiety diary in which patients can monitor their symptoms.

**Objective:**

The aims of this study were to provide insight into (1) usage intensity of the program (over time), (2) the course of symptoms during the 9 months of the study, and (3) the association between usage intensity and the course of symptoms.

**Methods:**

The Guided E-healTh for RElapse prevention in Anxiety and Depression (GET READY) program was guided by 54 MHPs working in primary care practices. Patients in remission from anxiety and depressive disorders were included. Demographic and clinical characteristics, including anxiety and depressive symptoms, were collected via questionnaires at baseline and after 3, 6, and 9 months. Log data were collected to assess the usage intensity of the program.

**Results:**

A total of 113 patients participated in the study. Twenty-seven patients (23.9%) met the criteria for the minimal usage intensity measure. The core modules were used by ≥70% of the patients, while the optional modules were used by <40% of the patients. Usage decreased quickly over time. Anxiety and depressive symptoms remained stable across the total sample; a minority of 15% (12/79) of patients experienced a relapse in their anxiety symptoms, while 10% (8/79) experienced a relapse in their depressive symptoms. Generalized estimating equations analysis indicated a significant association between more frequent face-to-face contact with the MHPs and an increase in both anxiety symptoms (β=.84, 95% CI .39-1.29) and depressive symptoms (β=1.12, 95% CI 0.45-1.79). Diary entries and the number of completed modules were not significantly associated with the course of symptoms.

**Conclusions:**

Although the core modules of the GET READY program were used by most of the patients and all patients saw an MHP at least once, usage decreased quickly over time. Most patients remained stable while participating in the study. The significant association between the frequency of contact and the course of symptoms most likely indicates that those who received more support had more symptoms, and thus, it is questionable whether the support offered by the program was sufficient to prevent these patients from relapsing.

**International Registered Report Identifier (IRRID):**

RR2-10.1186/s12888-019-2034-6

## Introduction

Despite effective treatments for anxiety and depressive disorders [1,2], maintenance phase relapse rates are high. Indeed, up to 57% of remitted patients experience a relapse of either their index disorder or another anxiety or depressive disorder within 4 years of remission [3]. Hence, relapse prevention is crucial in the maintenance phase. Having access to a relapse prevention program could help patients recognize early warning signs of relapse and take appropriate actions to prevent full relapse. There are several relapse prevention programs currently available for patients with remitted anxiety or depressive disorders. Previous research on patients with a depressive disorder showed that psychological relapse prevention programs reduce both residual symptoms [4,5] and relapse rates by 36% compared to treatment-as-usual [6]. Most relapse prevention programs solely involve face-to-face (FTF) contact, but programs using web-based formats are increasingly available [7]. Although web-based programs have the advantage of being easily accessible and flexible [8], the majority of them have low usage and high attrition rates [9-11]. This potentially undermines their effectiveness. A possible limitation of existing relapse prevention programs is that they are not explicitly based on patients’ preferences; taking these preferences into account can increase acceptance and adherence, which, in turn, enhances their effectiveness [12].

In the Netherlands, relapse prevention is provided by mental health professionals (MHPs) in primary care practices. However, many MHPs are unfamiliar with relapse prevention interventions, and the tools to support MHPs in providing relapse prevention are lacking [13]. Therefore, we developed the blended relapse prevention program “Guided E-healTh for RElapse prevention in Anxiety and Depression” (GET READY), which is explicitly based on patients’ preferences. The program aimed to prevent relapsing by promoting self-management skills. Patients’ preferences were obtained via a “discrete choice experiment,” in which a set of tasks comprising alternative hypothetical treatment options could be chosen by participants [14]. Patients preferred a relapse prevention program that included regular contact with a professional, flexible time investment based on their needs, and a personalized prevention plan. The purpose of the GET READY intervention was to provide a flexible program that could be used over a longer period depending on the symptom level of the patient. Based on these preferences, the GET READY program includes (1) regular FTF contact with an MHP, (2) web-based modules based on evidence-based (cognitive behavioral) interventions, divided into 2 core modules (including a personalized relapse prevention plan) and 12 optional modules, and (3) a mood and anxiety diary to monitor symptoms. Depending on the symptom level and needs of the patient, the program can be used over a longer period. This study examined (1) usage intensity of the program (over time), (2) the course of symptoms during the 9 months of the study, and (3) the association between usage intensity and the course of symptoms.

## Methods

### Design

The GET READY study was a pre-post study for remitted patients with an anxiety or depressive disorder [15]. This paper presents the results pertaining to the usage intensity of the GET READY program, the course of symptoms (at baseline and after 3, 6, and 9 months), and the association between usage intensity and the course of symptoms.

### Setting

This study was conducted in 50 primary care practice settings across the Netherlands. In the Netherlands, most primary care physicians (PCPs) employ an MHP (ie, nurse, psychologist, or social worker) who provides support and treatment for patients with mild mental health problems. These MHPs were involved in the GET READY program. Alternatively, for those patients whose MHP was not participating in the study, the program was offered via an ambulatory mental health care center. Patients began with an FTF meeting with an MHP, whereby they started composing a personalized relapse prevention plan. Next, patients could access web-based modules and a weekly diary via their computer, tablet, or smartphone. They were able to send messages to their MHP, ask for feedback on completed modules, and schedule FTF meetings with their MHP. All MHPs received a 4-hour training course, in which background information on relapse prevention, strategies for relapse prevention, and practical advice on using the program was provided [15]. PCPs did not play an active role in the study, although some MHPs regularly discussed patients with the PCP in their primary care practice.

### Participants

Patients were eligible to participate if they had received treatment in specialized mental health care centers for anxiety or depressive disorder in the previous 2 years. After receiving acute phase treatment, they were referred to primary care services. They had to be in full or partial remission according to their MHP or clinician (clinical judgment), have scored 50 or higher on the Global Assessment of Functioning scale [16], be at least 18 years old, and be sufficiently fluent in Dutch. Patients were excluded if they were participating in another structured psychological intervention, had no access to the internet, or still received specialized treatment for a comorbid psychiatric disorder. Maintenance antidepressant use was allowed.

### Procedures

We sought to recruit 50 MHPs and 126 patients for this study. Sample size calculations have been described elsewhere [15]. MHPs and patients were recruited from April 2017 to November 2018. Fifty-four MHPs working in primary care practices throughout the Netherlands were recruited via telephone, letters, advertisements on MHP websites, and through the researchers’ professional networks. Informed consent was obtained from MHPs at the start of the training course. PCPs had to agree that MHPs participated in the GET READY study. Patients were recruited either by their MHP or by their clinician at the end of their treatment (N=113), who provided brief information about the study. If patients were interested in participating, then the MHP or clinician asked consent from the patient to provide their contact details to the researchers. Next, consenting patients were contacted by the researchers and received additional information. Informed consent was obtained prior to administering the baseline questionnaire. This questionnaire assessed whether patients met the inclusion criteria pertaining to remission by administering the Inventory of Depressive Symptomatology Self-Report (IDS-SR) and the Beck Anxiety Inventory (BAI). Remission was defined as a score of <39 on the IDS-SR and a score of <30 on the BAI. Scores above these cutoff points indicate severe symptoms that require additional treatment to relapse prevention [17,18]. Therefore, patients with a score of ≥39 on the IDS-SR or ≥30 on the BAI were excluded from the study.

### Ethical Approval

The Medical Ethics Committee of the Vrije Universiteit University Medical Center Amsterdam deemed that ethical approval was not required according to Dutch legislation (registration 2016.280) and thus gave their permission to conduct the study.

### GET READY Program

The central aim of the program was to prevent relapse via the promotion of self-management skills. In the field of mental health, strengthening self-management skills is increasingly important, insofar as it allows patients to self-manage their own mental health [19]. More information regarding the content of the GET READY program has been published previously [15]. The program comprised several components. The program offered both FTF and web-based contact with an MHP. Every patient had at least 1 FTF engagement at the start of the study, and patients and MHPs were encouraged by the researchers to have FTF contact every 3 months. In addition, patients were encouraged to contact MHPs if their symptoms increased. In the FTF contact between patients and MHPs, usage of the program was discussed, and patients were encouraged to use the relapse prevention plan and to complete the diary and web-based modules. Patients were able to request feedback from their MHP when using the web-based modules. Besides the feedback on specific modules, patients and MHPs could also send and receive messages via the web-based platform. MHPs had access to their patients’ data and could check whether they had logged in or if they had completed modules and the weekly diary. In the event that a patient did not complete a module within a week, they were sent an automatic reminder.

**Figure 1:**
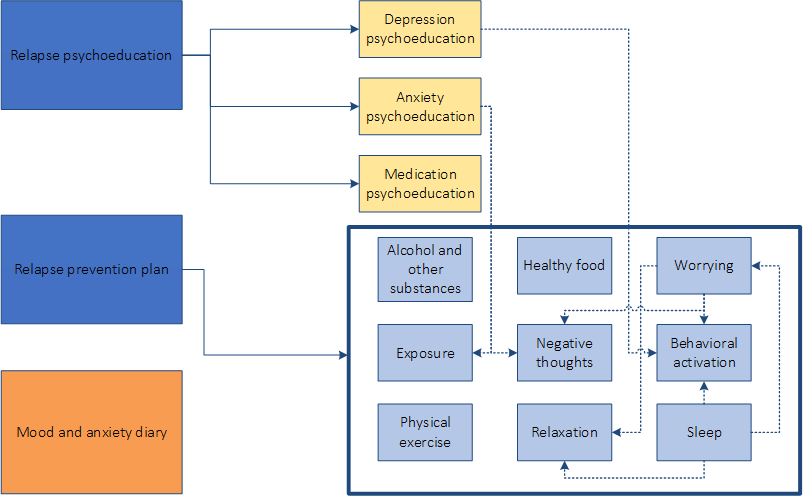
Overview of eHealth modules.

The web-based modules were divided into 2 core modules “relapse psychoeducation” and “relapse prevention plan” (see Multimedia Appendix 1) and 12 optional modules, which included 3 psychoeducation modules with information on depression, anxiety, and medication. The other 9 optional modules contained information on specific topics such as exposure, negative thoughts, and sleep (see Figure 1). These modules also contained exercises, videos, and examples of fictive patients. Some modules had overlapping themes, and patients could easily open these linked modules from the other module (see the dotted lines in Figure 1). Finally, the GET READY program included a “mood and anxiety diary,” which allowed patients to monitor their symptoms. Patients received weekly reminders to complete the diary. When patients logged in for the first time, the core components “relapse psychoeducation,” “relapse prevention plan,” and the “mood and anxiety diary” were available. If patients completed the “relapse psychoeducation” module, the “depression/anxiety/medication psychoeducation” modules were automatically set up. Likewise, if patients completed the “relapse prevention plan,” they could choose which optional modules they wish to complete based on their preferences and goals.

### Data Collection

Patients were invited to complete questionnaires at baseline (T0) and after 3 (T1), 6 (T2), and 9 months (T3). Completion of the questionnaires took 20-30 minutes. If necessary, patients received an email reminder after 1 week. As part of the treatment protocol, patients were also prompted to complete the mood and anxiety diary once a week for a period of 9 months (39 times). MHPs were requested to complete a case registration form after each FTF contact, in which the clinical status of patients and the duration and content of the FTF contacts were described. In order to assess the usage intensity of the program, log data from the web-based platform were collected.

### Measures

#### Demographic and Clinical Variables

Demographic and clinical variables of patients were assessed at baseline using the questionnaire. Moreover, in the baseline questionnaire, patients were asked to score their own perceived risk of relapse as well as their expectations about the effectiveness of the relapse prevention program (0%-100%). Anxiety severity was measured using the BAI, and symptoms in the past week were assessed. This questionnaire contains 21 items, all of which are rated on a 0- to 3-point scale, with a total score ranging from 0 to 63, with ≥30 indicating severe anxiety symptoms [20]. Severity of depression was measured using the IDS-SR [21]. Depressive symptoms in the past week were assessed. This questionnaire contains 30 items, all of which are rated on a 0- to 3-point scale, and when adding up 28 of the 30 items, the total score ranged from 0 to 84, with ≥39 indicating severe depressive symptoms [18]. To provide insight into the baseline clinical characteristics of patients, anxiety sensitivity and general functioning and disability were also measured. Anxiety sensitivity was measured using the Anxiety Sensitivity Index [22]. This questionnaire has 16 items, all of which are rated on a 0- to 4-point scale, with a total score ranging from 0 (no anxiety sensitivity) to 64 (severe anxiety sensitivity). General functioning and disability were measured using the World Health Organization Disability Assessment Schedule 2.0. This questionnaire has 36 items, all of which are rated on a 0- to 4-point scale, with a total score ranging from 0 (no disability) to 100 (full disability) [23]. Medication and health care use was measured using the Trimbos/iMTA Questionnaire for Costs Associated with Psychiatric Illness (TiC-P) [24].

#### Primary Outcome

##### Program Usage Variable

Log data from the web-based platform was used to assess the web-based usage intensity of the program. This included the number of messages from patients to MHPs or vice versa, the number of completed modules, and the number of diary entries. The frequency of FTF contact between patients and MHPs was registered with the TiC-P [24]. Participants were divided into low and regular users based on the median of the separate usage variables, as the data was nonnormally distributed. If participants had completed at least the median amount of FTF contact with the MHP (median 1), modules (median 4), and diary entries (median 4), then they were classified as regular users of these specific usage variables. If they completed less than the median of the separate usage variables, then they were considered to be low users. Furthermore, a “minimal usage intensity” measure was composed. If patients had at least 1 FTF contact during the intervention period, completed the core components of the program (relapse psychoeducation module, relapse prevention plan, at least 4 mood and anxiety diary entries), and completed at least 1 extra module, then they were classified as regular users. If they did not complete these components, they were considered to be low users.

##### Course of Symptoms

To explore the course of symptoms during the study, the severity of anxiety and depressive symptoms was measured at baseline and 3-month intervals (T1, T2, T3) using the BAI and the IDS-SR. Deterioration/relapse was defined as an increase of at least 1 SD on the IDS-SR or of an increase on the BAI between T0 and T3. If there was an increase of 1 SD on the IDS-SR and the BAI, this was also regarded as deterioration/relapse. Similarly, symptom improvement was defined as a decrease of at least 1 SD. The SD was calculated using data from the Netherlands Study of Depression and Anxiety [25], the study of Kok et al [5], and this study, resulting in an SD of 9.3 on the IDS-SR and an SD of 6.6 on the BAI. By approaching the definition of relapse this way, patients can be regarded as their own controls, and an increase of 1 SD most likely indicates a clinically significant increase in symptoms, and thus indicate relapse.

### Statistical Analysis

**Figure 2:**
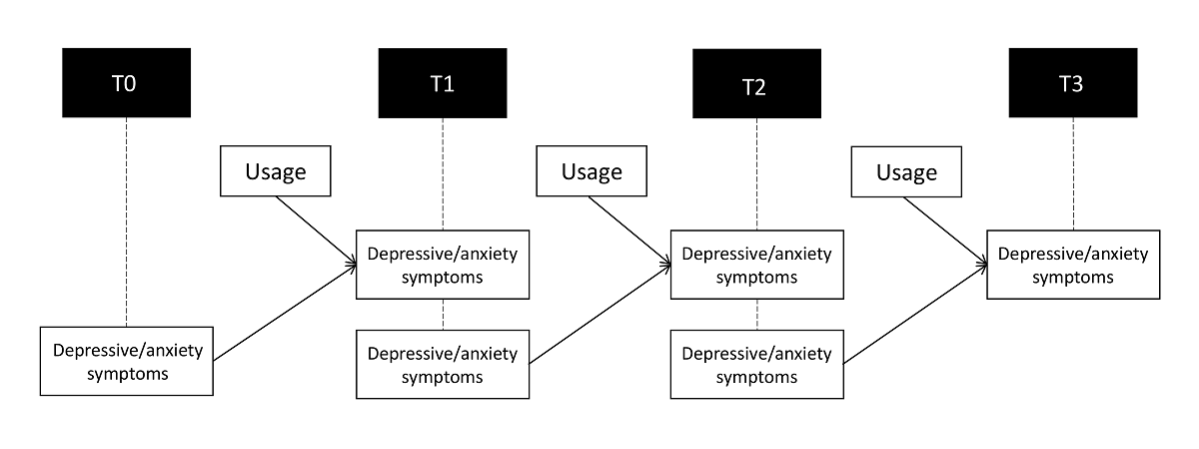
Time-lag model. T0: baseline assessment; T1: assessment after 3 months; T2: assessment after 6 months; T3: assessment after 9 months.

Descriptive statistics were used to describe the characteristics of the participants to illustrate the extent to which patients used the program (over time) and to explore the course of symptoms. Explorative analyses were conducted to study the association between usage intensity and the course of symptoms. The course of symptoms was determined for both regular and low users in accordance with the “minimal usage intensity” measure as well as for the separate usage intensity measures. Differences in anxiety and depressive symptoms between regular and low users were tested using the Mann-Whitney *U* test (as these were nonnormally distributed), while Bonferroni corrections were applied to correct for multiple testing [26]. Generalized estimating equations (GEE) analyses were carried out to examine the longitudinal association between the different usage intensity variables and the course of symptoms. The usage intensity variables indicated usage of the program between baseline and T1, T1 and T2, and T2 and T3. In this way, the association between usage intensity and the course of symptoms at the point of each follow-up questionnaire was assessed by taking into account the usage intensity in the period immediately prior to the follow-up questionnaire. A time-lag model was used, in which an adjustment was made for the outcome at time point *t* – 1, as it assumed that the outcome at time *t* was predicted by the outcome at time *t* – 1 (see Figure 2). All data analyses were performed using SPSS 26 (IBM Corp). Further details regarding the methods employed in this study can be found in the study protocol [15].

## Results

### Participant Characteristics

The demographic and clinical characteristics of the participants (N=113) are reported in Table 1. The mean age at baseline was 43 (SD 12.9) years. More than half of the participants were females (65/113, 57.5%), while more than half of the participants attended higher professional education or university (64/113, 56.6%). Overall, 36.3% (41/113) of the participants reported being treated for a depressive disorder, 23.9% (27/113) stated they had been treated for an anxiety disorder, and 39.8% (45/113) stated they had been treated for both.

**Table 1:** Demographic and clinical characteristics of the total sample (N=113).

Variables			Value
**Demographic variables**			
	Age (years), mean (SD)		42.9 (12.9)
	Sex (female), n (%)		65 (57.5)
	Nationality (Dutch), n (%)		105 (92.9)
	**Marital status, n (%)**		
		Single	45 (39.8)
		In relationship	68 (60.2)
	**Highest educational level, n (%)**		
		High school	23 (20.3)
		Secondary vocational education	22 (19.5)
		Higher professional education or university	64 (56.6)
		Unknown	4 (3.6)
	**Occupational, n (%)**		
		Employed	79 (69.9)
		Sick leave	18 (15.9)
		Other	16 (14.2)
**Clinical variables**			
	**Clinical history, n (%)**		
		Treatment for depressive disorder	41 (36.3)
		Treatment for anxiety disorder	27 (23.9)
		Treatment for both depressive disorder and anxiety disorder	45 (39.8)
	Number of times received treatment for mental health problems, mean (SD)		3.5 (3.3)
	Time passed since referral back to the primary care physician from specialized care (months), mean (SD)		5.9 (6.3)
	Age of first onset (years), mean (SD)		27.6 (13.8)
	Positive family history of anxiety or depressive disorder, n (%)		60.0 (53.1)
	Anxiety sensitivity, mean (SD)		10.7 (7.9)
	General functioning and disability, mean (SD)		23.6 (15)
	Anxiety severity, mean (SD)		10.2 (6.6)
	Depression severity, mean (SD)		20.6 (9.5)

### Usage Intensity

The use of the program is described in 3 subcategories: (1) contact with MHP, (2) completed modules, and (3) diary entries.

#### Contact With MHP

The option to correspond with MHPs via the web-based platform was rarely exercised by participants. In total, the 113 patients sent 157 messages to their MHPs (median 0 [IQR 0.0-2.0]) and received 260 messages in return from their MHPs (median 1.0 [IQR 0.0-3.0]). Sixty-five patients (57.5%) never sent a message to their MHP, and 45 patients (39.8%) never received a single message from their MHP. All participants had initial FTF contact with their MHPs. During the 9 months of the study, there were 260 FTF follow-up meetings (median 1.0 [IQR 0.0-4.0]). Forty-nine participants (43.4%) did not have any follow-up meetings with their MHP. Forty-one participants (36.3%) met their MHP at least every 3 months, as prescribed in the research protocol. The number of FTF appointments ranged from 0 to 13.

#### Completed Modules

A median of 4 modules were completed by the participants (IQR 2.0-8.0). Of the 113 participants, 1 (0.01%) completed all the 14 available modules, while 17 participants (15%) failed to complete any module. The 2 core modules were completed the most: 74.3% (84/113) of the participants completed the module “relapse psychoeducation” and 69.9% (79/113) completed the module “relapse prevention plan.” Approximately 46%-54% of the patients completed the other 3 psychoeducation modules, while less than 40% of patients completed the optional modules.

#### Diary Entries

**Figure 3:**
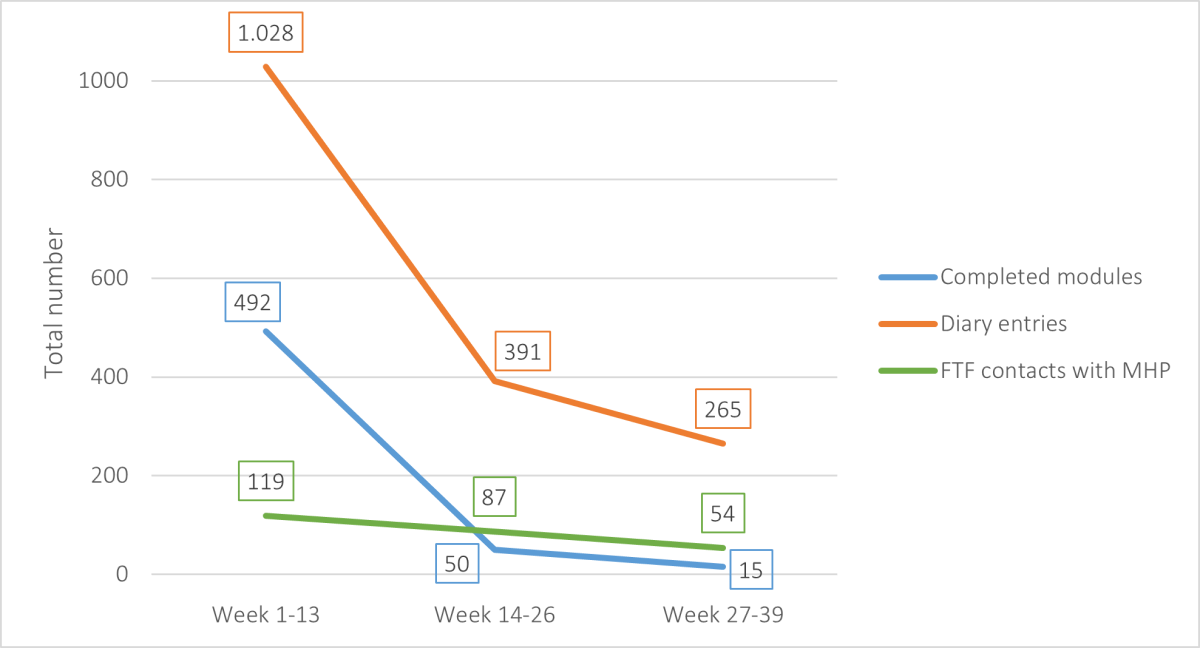
Usage of the modules, diary, and face-to-face contacts over time. FTF: face-to-face; MHP: mental health professional.

The number of diary entries varied substantially across the participants, ranging from 0 to 159, with a median of 4 (IQR 1.0-15.0). Seventeen participants (15%) never reported on their mood and anxiety. Only 12 participants (10.6%) completed the diary weekly for the entire duration of the study. Usage of the program decreased considerably over time, as can be seen in Figure 3. In particular, there was a strong decrease in both the number of completed modules and number of diary entries. The median for when participants completed their last module was 31 (IQR 10.0-92.5) days after registering on the web-based platform.

### Course of Symptoms

In the overall sample, anxiety and depressive symptoms decreased slightly over time. For all 113 participants, the mean BAI score at baseline was 10.2 (SD 6.6). After 9 months, the mean BAI score of the remaining 79 participants was 9.3 (SD 8.2). The differences over time were not significant, as indicated by the overlapping error bars in Figure S1 in Multimedia Appendix 2. A completer analysis (which included only those patients who completed T3) produced similar results. The mean IDS-SR score of all 113 participants decreased from 20.6 (SD 9.5) at baseline to 17.3 (SD 11.8) at 9 months (T3). The differences over time were not significant, as indicated by the overlapping error bars in Figure S2 in Multimedia Appendix 2. A completer analysis produced similar results. Regarding changes in symptomatology (stable/deteriorated/improved), for anxiety symptoms, it was found that the majority of the 79 patients who completed T3 remained stable over time (52/79, 66%), 12 patients (15%) experienced a deterioration of at least 1 SD (defined as a relapse), and 15 patients (19%) saw their anxiety symptoms improve. The numbers were comparable for depression symptoms: 53 patients (67%) remained stable, 8 patients (10%) deteriorated/relapsed, and 18 patients’ (23%) depressive symptoms improved. Seven patients (9%) experienced a deterioration in both their anxiety and depressive symptoms. For all 3 categories (stable/deteriorated/improved), antidepressant medication use remained largely stable. For depressive symptoms, most patients in the stable and deterioration group used antidepressant medication: 57% (30/53) and 75% (6/8) respectively. In the improved group, 50% (9/18) used antidepressant medication. For anxiety symptoms, the majority of patients in the stable and deterioration group used antidepressant medication: 64% (33/52) and 58% (7/12) respectively. In the improved group, 33% (5/15) used antidepressant medication.

### Association Between Usage Intensity and Course of Symptoms

#### Minimal Usage Intensity Measure

Of the 113 patients, 27 (23.9%) met the criteria for the “minimal usage intensity” measure, and hence, these patients were defined as regular users. Figure S3 in Multimedia Appendix 2 depicts the course of anxiety symptoms for both regular and low users, as measured by the combined “minimal usage intensity” measure. No significant differences were found in the anxiety symptoms between regular and low users (T0: *U*=1122.0, *P*=.79; T1: *U*=840.0, *P*=.52; T2: *U*=738.5, *P*=.79; T3: *U*=623.5, *P*=.59). Figure S4 in Multimedia Appendix 2 depicts the course of depressive symptoms for both regular and low users. Although the mean scores for regular users were higher across all the time points, no statistically significant differences were found between low and regular users (T0: *U*=1025.0, *P*=.36; T1: *U*=700.0, *P*=.07; T2: *U*=708.0, *P*=.57; T3: *U*=506.5, *P*=.08). The number of appointments in specialized mental health care facilities during the study did not significantly differ between regular and low users (T1: *U*=909.0, *P*=.92; T2: *U*=702.0, *P*=.39; T3: *U*=631.0, *P*=.55). There was a significant difference between regular and low users in terms of the number of appointments they had with a psychologist or psychiatrist in a private practice at T3 (*U*=575.0, *P*=.04), that is, low users had more appointments than regular users. However, after applying the Bonferroni correction, this difference was no longer significant. No significant differences in medication use between regular and low users were found (T0: *U*=1148.0, *P*=.92; T1: *U*=900.5, *P*=.87; T2: *U*=727.5, *P*=.66; T3: *U*=645.0, *P*=.71).

#### Separate Usage Intensity Measures

The mean BAI scores and IDS-SR scores for regular users (median use of usage variable or higher) and low users (below median of usage variable) on the separate usage intensity measures across all 4 time points are shown in Multimedia Appendix 3. Patients who had 1 or more FTF meetings with their MHP after the initial FTF contact experienced a higher score on the BAI and IDS-SR at T3. For diary entries and the number of completed modules, the BAI and IDS-SR scores did not differ between regular and low users.

#### GEE Analyses

In the GEE analyses, all of the separate usage variables were used to model the course of anxiety and depressive symptoms in a multivariate analysis. GEE analyses indicated no significant association between module completion and number of diary entries and the course of anxiety or depressive symptoms (Table 2). A significant association was found between the frequency of FTF contact with MHPs and the course of anxiety and depressive symptoms. The coefficient of .84 (95% CI .39-1.29) indicates that each additional FTF meeting with an MHP was associated with an increased BAI score of .84 in the next measurement (corrected for the BAI score one measurement prior). Similarly, the coefficient of 1.12 (95% CI .45-1.79) indicates that each additional FTF meeting with an MHP was associated with an increased IDS-SR score of 1.12 in the next measurement (corrected for the IDS-SR score one measurement prior). Therefore, more FTF contact with MHPs was significantly associated with higher anxiety and depressive scores.

**Table 2:** Generalized estimating equations analysis of the longitudinal associations of separate usage intensity variables with anxiety and depressive symptoms.

	Anxiety symptoms			Depressive symptoms		
	β (SE)	95% CI	*P* value^a^	β (SE)	95% CI	*P* value
Module completion	.12 (.13)	–.15 to .38	.39	.06 (.12)	–.18 to .30	.65
Face-to-face contact with mental health professional	.84 (.23)	.39 to 1.29	*<.001*	1.12 (.34)	.45 to 1.79	*.001*
Diary completion	–.05 (.03)	–.10 to .003	.07	–.04 (.03)	–.09 to .02	.19

^a^*P* values <.05 were considered to indicate significance and are shown in italics*.*

## Discussion

### Principal Results

This study has shown the usage intensity of the GET READY relapse prevention program, explored the course of symptoms of participants across the duration of the study, and examined the association between usage intensity and the course of symptoms. The core modules were used by ≥70% of the patients, while optional modules were regarded as elective and used as such (<40% of the patients). Of the 113 patients, 27 (23.9%) were defined as regular users according to the minimal usage intensity measure. Usage of the self-management components of the program (the web-based modules and web-based mood and anxiety diary) decreased quickly over time. Although no causal effect of the GET READY intervention on the severity of psychopathology could be established owing to its pre-post design, it appeared that most patients remained stable or experienced symptom improvement while they engaged with the GET READY program. Having more FTF contact with MHPs was significantly associated with an increase in anxiety and depressive symptoms. The other usage intensity variables were not significantly associated with the course of symptoms. Overall, the participants were highly educated and employed. These results are consistent with those reported in other studies on web-based or eHealth interventions [27,28], which have shown that this group is more likely to use web-based interventions. Similarly to Kontos et al [28], we also found it difficult to access patients with lower educational levels, which is problematic given that this program might also be beneficial for this group. Therefore, future relapse prevention studies should attempt to access participants with lower educational levels by seeking input from this group during the developmental phase of interventions.

The core components of the program were used fairly well, as the 2 core modules “relapse psychoeducation” and “relapse prevention plan” were completed by 74.3% (84/113) and 69.9% (79/113) of patients, respectively. As expected, optional modules were used less frequently than the core modules, with less than 40% of patients completing them. This result is consistent with data from Hollandäre et al [29], who showed that their basic modules were used more often than optional modules. Nonetheless, the usage of the optional modules in this study was relatively low in comparison to that in other studies [30-33]. The average usage intensity in these other programs was higher, with around 50% of the participants completing all available modules. However, these studies varied in terms of the number of modules (n=3-12), not to mention that the web-based programs were not focused on relapse prevention but rather on treating anxiety and depressive disorders. In this study, patients had already finished treatment for anxiety or depressive disorders, were in remission, and therefore may not have felt the need to actively engage in a relapse prevention program. One explanation for the relatively low usage could be simply that the patients experienced treatment fatigue [34]. Another explanation might be that since all the patients had received treatment prior to this study, the lessons learned from treatment were very much at the forefront of their mind and, as such, they felt no desire to repeat these lessons in the relapse prevention program. Indeed, it appeared that patients selected the modules that applied to their situation. To conclude, the optional modules in our relapse prevention program were regarded as elective and used as such.

Usage of the program decreased rapidly over time, as most patients used the program for a median of 1 month after registering on the web-based platform. Although this finding has been reported in previous studies on (web-based) guided self-help programs [10,11], it was contrary to the aim of the intervention, which was to provide a flexible program that can be used over a longer period of time depending on patients’ symptom levels. Prior studies demonstrated that both the absence of symptoms and an increase of symptoms might hinder patients’ capacity to actively use a program. Potentially, patients with fewer symptoms may not need to engage with the entire program to feel well again and cease using the program after obtaining the benefits [35,36]. At the same time, a qualitative study on the GET READY program indicated that increased symptom levels might also limit patients from further using the program [37]. An increase in depressive or anxiety symptoms may result in avoidance behavior, which may also lead to avoiding actively working on the web-based modules. This underlines the importance of the proactive role to be played by MHPs in terms of stimulating patients who are vulnerable to relapsing to continue using the program. Another important way of keeping patients engaged might be the further personalization of the intervention content, for example, by increasing the depth of tailored feedback, providing real-time feedback, and customizing the content based on current symptoms [9,37].

Although all of the patients were in remission, they nevertheless appeared vulnerable to relapse: patients had already received an average of 3.5 treatments in specialized care, 53.1% (60/113) had a family history of anxiety/depression, and 39.8% (45/113) had received treatment for both an anxiety and a depressive disorder, while their baseline mean symptom levels showed mild residual anxiety and depressive symptoms. In the overall sample, anxiety and depressive symptoms decreased slightly over time. Most participants remained stable, while 19%-23% of patients experienced symptom improvement. Only 10%-15% of the patients experienced a relapse. In comparison to other studies, our results show lower relapse rates [38,39]. Hardeveld et al [38] found that after 10 months, 20%-30% of patients experienced a relapse of their depressive symptoms. Similarly, Taylor et al [39] found that around 30% of patients experienced a relapse of their anxiety symptoms within a year. Although no causal pathway could be established in this pre-post study, these results nevertheless indicate that the GET READY program could potentially protect patients from relapse. However, as the definition of relapse differs across studies, comparing the results can be difficult. Therefore, efforts should be made in the field to reach a consensus regarding the definition and assessment of relapse in depression and anxiety disorders. Moreover, the effectiveness of the GET READY program in preventing relapse should be tested in a randomized controlled trial (RCT).

Patients who experienced a deterioration in symptoms more often used antidepressant medication than patients whose symptoms improved. However, this result should be interpreted with caution, as no causal pathway can be established. This study design is not feasible to investigate the influence of medication on the course of symptoms. Patients who had more FTF contact with MHPs had significantly higher anxiety and depressive scores than patients who had less FTF contact with MHPs. It is questionable whether the support they received by their MHP was sufficient to engender a subsequent decrease in symptoms. At the same time, this result might indicate that the web-based program in itself does not provide enough support to patients who experience a deterioration of symptoms. As aforesaid, this is a pre-post study; therefore, no causal pathway could be established [40]. An alternative explanation for this significant association might be that patients adequately responded to early symptoms of relapse by reaching out to their MHP. This explanation is in line with an earlier study, which showed that patients with more severe symptoms were more likely to receive help [41]. Furthermore, no evidence was found for an association between the number of completed modules and diary entries on the one hand and the course of symptoms on the other hand. Although no comparison with other relapse prevention programs is currently possible, other treatment studies have found that more completed web-based modules are associated with better anxiety and depression outcomes [31,42]. The same applies to the number of diary entries [43]. When comparing this study to these studies, it becomes apparent that the sample size of these studies was larger. Therefore, as well as the difference in population (remitted vs present disorder) and aims of the program (relapse prevention vs treatment), these studies may also have had more scope to detect an association.

### Limitations

There are several potential limitations in this study. First, attrition from the study was relatively high, with only 79 (69.9%) of the 113 participants completing the last follow-up questionnaire. Despite this, the statistical methods applied in this study, especially GEE analyses, are expedient for handling missing data. Second, self-selection bias and the fact that the patients were highly educated might restrict the generalizability of the results. However, Donkin et al’s [44] study showed no indication that these factors actually limit the generalizability, as they found no evidence that these factors were related to study outcomes. Third, this study had a limited follow-up period of 9 months. A longer follow-up period of 2 years would have provided greater insight into the course of anxiety and depressive symptoms over a longer period of time, which, in turn would have facilitated better comparison with other studies [38,45]. However, for pragmatic reasons, it was not feasible to extend the follow-up period. Fourth, owing to methodological considerations, no time-to-event analysis could be performed, as the assumptions for this analysis could not be met. Finally, the definitions of “regular use” that were used in this study should be interpreted with caution. As the median of several usage intensity measures was relatively low (ie, FTF contact median=1), regular use could still indicate relatively low usage when compared to the intended amount of usage (ie, FTF contact once every 3 months=3 FTF meetings). However, to enhance readability we opted to use the terms “regular use” and “low use.”

### Implications for Practice and Research

This study highlights the importance of providing personalized and guided relapse prevention to remitted patients with anxiety and depressive disorders. Usage of the program decreased quickly over time, possibly indicating a rapid decrease in the motivation of patients. As aforesaid, this decrease in motivation can be explained by different causal factors. Therefore, MHPs have the important task of monitoring and motivating patients via personalized intervention strategies, thus ensuring that patients receive guidance when they need it the most. Further research in an RCT with a longer follow-up duration is necessary to establish the effectiveness of blended relapse prevention programs. Within the design of an RCT, greater insight can also be obtained into the association between usage intensity and the course of symptoms.

### Conclusions

When relapse prevention was offered, most patients used the core modules, while optional modules were completed by a smaller sample. As indicated in an earlier study [14], the patients showed that they preferred a low level of time investment for relapse prevention programs. Despite the relatively low usage and low time investment, most patients remained stable while participating in the GET READY study. Patients who had more FTF contact with their MHP experienced more anxiety and depressive symptoms. Owing to the pre-post design of this study, no causal pathway could be established. An RCT is needed to provide insight into the effectiveness of the GET READY program and to further explore the causal relationship between usage intensity and the course of symptoms.

## Appendix

Multimedia Appendix 1 Home page for patients with core modules, namely, relapse psychoeducation and relapse prevention plan, and the mood and anxiety diary.

Multimedia Appendix 2 Course of symptoms specified by usage intensity.

Multimedia Appendix 3 Low and regular use of separate usage intensity measures.

## Abbreviations

BAI: Beck Anxiety Inventory
FTF: face-to-face
GEE: generalized estimating equations
GET READY: Guided E-healTh for RElapse prevention in Anxiety and Depression
IDS-SR: Inventory of Depressive Symptomatology Self-Report
MHP: mental health professional
PCP: primary care physician
RCT: randomized controlled trial
TiC-P: Trimbos/iMTA Questionnaire for Costs Associated with Psychiatric Illness

## References

[1] Bandelow B, Sagebiel A, Belz M, Görlich Yvonne, Michaelis S, Wedekind D (2018). Enduring effects of psychological treatments for anxiety disorders: meta-analysis of follow-up studies. Br J Psychiatry.

[2] Cuijpers P (2018). The Challenges of Improving Treatments for Depression. JAMA.

[3] Scholten WD, Batelaan NM, Penninx BW, van Balkom Anton J L M, Smit JH, Schoevers RA, van Oppen P (2016). Diagnostic instability of recurrence and the impact on recurrence rates in depressive and anxiety disorders. J Affect Disord.

[4] Hoorelbeke K, Koster EHW (2017). Internet-delivered cognitive control training as a preventive intervention for remitted depressed patients: Evidence from a double-blind randomized controlled trial study. J Consult Clin Psychol.

[5] Kok G, Burger H, Riper H, Cuijpers P, Dekker J, van Marwijk H, Smit F, Beck A, Bockting CLH (2015). The Three-Month Effect of Mobile Internet-Based Cognitive Therapy on the Course of Depressive Symptoms in Remitted Recurrently Depressed Patients: Results of a Randomized Controlled Trial. Psychother Psychosom.

[6] Biesheuvel-Leliefeld K, Kok G, Bockting C, Cuijpers P, Hollon S, van Marwijk Harm W J, Smit F (2015). Effectiveness of psychological interventions in preventing recurrence of depressive disorder: meta-analysis and meta-regression. J Affect Disord.

[7] Hennemann S, Farnsteiner S, Sander L (2018). Internet- and mobile-based aftercare and relapse prevention in mental disorders: A systematic review and recommendations for future research. Internet Interv.

[8] Carlbring P, Andersson G, Cuijpers P, Riper H, Hedman-Lagerlöf E (2018). Internet-based vs. face-to-face cognitive behavior therapy for psychiatric and somatic disorders: an updated systematic review and meta-analysis. Cogn Behav Ther.

[9] Beatty L, Binnion C (2016). A Systematic Review of Predictors of, and Reasons for, Adherence to Online Psychological Interventions. Int J Behav Med.

[10] Eysenbach G (2005). The law of attrition. J Med Internet Res.

[11] Kelders SM, Bohlmeijer ET, Van Gemert-Pijnen JE (2013). Participants, usage, and use patterns of a web-based intervention for the prevention of depression within a randomized controlled trial. J Med Internet Res.

[12] Cuijpers P, van Straten A, Warmerdam L, van Rooy M (2010). Recruiting participants for interventions to prevent the onset of depressive disorders: possible ways to increase participation rates. BMC Health Serv Res.

[13] Hermens MLM, Muntingh A, Franx G, van Splunteren PT, Nuyen J (2014). Stepped care for depression is easy to recommend, but harder to implement: results of an explorative study within primary care in the Netherlands. BMC Fam Pract.

[14] Muntingh ADT, Hoogendoorn AW, Van Schaik DJF, Van Straten A, Stolk EA, Van Balkom AJLM, Batelaan NM (2019). Patient preferences for a guided self-help programme to prevent relapse in anxiety or depression: A discrete choice experiment. PLoS One.

[15] Krijnen-de Bruin E, Muntingh ADT, Hoogendoorn AW, van Straten A, Batelaan NM, Maarsingh OR, van Balkom AJLM, van Meijel B (2019). The GET READY relapse prevention programme for anxiety and depression: a mixed-methods study protocol. BMC Psychiatry.

[16] American Psychiatric Association (1994). DSM-IV Diagnostic and Statistical Manual of Mental Disorders.

[17] Beck A, Steer R (1993). Beck Anxiety Inventory Manual.

[18] Inventory of Depressive Symptomatology (IDS) and Quick Inventory of Depressive Symptomatology (QIDS) - Interpretation.

[19] Kemp V (2011). Use of ‘chronic disease self-management strategies’ in mental healthcare. Current Opinion in Psychiatry.

[20] Beck AT, Epstein N, Brown G, Steer RA (1988). An inventory for measuring clinical anxiety: psychometric properties. J Consult Clin Psychol.

[21] Rush AJ, Gullion CM, Basco MR, Jarrett RB, Trivedi MH (1996). The Inventory of Depressive Symptomatology (IDS): psychometric properties. Psychol Med.

[22] Reiss S, Peterson RA, Gursky DM, McNally RJ (1986). Anxiety sensitivity, anxiety frequency and the prediction of fearfulness. Behav Res Ther.

[23] WHO disability assessment schedule 2.0. WHO.

[24] Hakkaart-van Roijen L, van Straten A, Tiemens B, Donker M (2002). Trimbos/iMTA questionnaire for costs associated with psychiatric illness (TiC-P). Research Gate.

[25] Penninx BW, Beekman AT, Smit JH, Zitman FG, Nolen WA, Spinhoven P, Cuijpers P, De Jong PJ, Van Marwijk HW, Assendelft WJ, Van Der Meer K, Verhaak P, Wensing M, De Graaf R, Hoogendijk WJ, Ormel J, Van Dyck R, NESDA Research Consortium (2008). The Netherlands Study of Depression and Anxiety (NESDA): rationale, objectives and methods. Int J Methods Psychiatr Res.

[26] Armstrong RA (2014). When to use the Bonferroni correction. Ophthalmic Physiol Opt.

[27] Torrent-Sellens J, Díaz-Chao Ángel, Soler-Ramos I, Saigí-Rubió Francesc (2016). Modelling and Predicting eHealth Usage in Europe: A Multidimensional Approach From an Online Survey of 13,000 European Union Internet Users. J Med Internet Res.

[28] Kontos E, Blake KD, Chou WS, Prestin A (2014). Predictors of eHealth usage: insights on the digital divide from the Health Information National Trends Survey 2012. J Med Internet Res.

[29] Holländare F, Johnsson S, Randestad M, Tillfors M, Carlbring P, Andersson G, Engström I (2011). Randomized trial of Internet-based relapse prevention for partially remitted depression. Acta Psychiatr Scand.

[30] Donkin L, Hickie IB, Christensen H, Naismith SL, Neal B, Cockayne NL, Glozier N (2013). Rethinking the dose-response relationship between usage and outcome in an online intervention for depression: randomized controlled trial. J Med Internet Res.

[31] Hilvert-Bruce Z, Rossouw PJ, Wong N, Sunderland M, Andrews G (2012). Adherence as a determinant of effectiveness of internet cognitive behavioural therapy for anxiety and depressive disorders. Behav Res Ther.

[32] Van Gemert-Pijnen Julia Ewc, Kelders SM, Bohlmeijer ET (2014). Understanding the usage of content in a mental health intervention for depression: an analysis of log data. J Med Internet Res.

[33] van Straten Annemieke, Cuijpers P, Smits N (2008). Effectiveness of a web-based self-help intervention for symptoms of depression, anxiety, and stress: randomized controlled trial. J Med Internet Res.

[34] Heckman BW, Mathew AR, Carpenter MJ (2015). Treatment Burden and Treatment Fatigue as Barriers to Health. Curr Opin Psychol.

[35] Neil AL, Batterham P, Christensen H, Bennett K, Griffiths KM (2009). Predictors of adherence by adolescents to a cognitive behavior therapy website in school and community-based settings. J Med Internet Res.

[36] Postel MG, de Haan Hein A, ter Huurne Elke D, Becker ES, de Jong Cor A J (2010). Effectiveness of a web-based intervention for problem drinkers and reasons for dropout: randomized controlled trial. J Med Internet Res.

[37] Krijnen-de Bruin E, Geerlings JA, Muntingh AD, Scholten WD, Maarsingh OR, van Straten A, Batelaan NM, van Meijel B (2021). Evaluation of a Blended Relapse Prevention Program for Anxiety and Depression in General Practice: Qualitative Study. JMIR Form Res.

[38] Hardeveld F, Spijker J, De Graaf Ron, Hendriks S, Licht C, Nolen W, Penninx B, Beekman A (2013). Recurrence of major depressive disorder across different treatment settings: results from the NESDA study. J Affect Disord.

[39] Taylor JH, Jakubovski E, Bloch MH (2015). Predictors of anxiety recurrence in the Coordinated Anxiety Learning and Management (CALM) trial. J Psychiatr Res.

[40] Thiese MS (2014). Observational and interventional study design types; an overview. Biochem Med (Zagreb).

[41] ten Have M, de Graaf R, Vollebergh W, Beekman A (2004). What depressive symptoms are associated with the use of care services?. Journal of Affective Disorders.

[42] Christensen H, Griffiths KM, Korten A (2002). Web-based cognitive behavior therapy: analysis of site usage and changes in depression and anxiety scores. J Med Internet Res.

[43] de Graaf L Esther, Huibers MJH, Riper H, Gerhards SAH, Arntz A (2009). Use and acceptability of unsupported online computerized cognitive behavioral therapy for depression and associations with clinical outcome. J Affect Disord.

[44] Donkin L, Hickie IB, Christensen H, Naismith SL, Neal B, Cockayne NL, Glozier N (2012). Sampling bias in an internet treatment trial for depression. Transl Psychiatry.

[45] Bockting C, Klein N, Elgersma H, van Rijsbergen Gd, Slofstra C, Ormel J, Buskens E, Dekker J, de Jong Pj, Nolen W, Schene A, Hollon S, Burger H (2018). Effectiveness of preventive cognitive therapy while tapering antidepressants versus maintenance antidepressant treatment versus their combination in prevention of depressive relapse or recurrence (DRD study): a three-group, multicentre, randomised controlled trial. The Lancet Psychiatry.

