# Supplementary material for: Usage Intensity of a Relapse Prevention Program and Its Relation to Symptom Severity in Remitted Patients With Anxiety and Depression: Pre-Post Study
Source: JMIR Ment Health. 2022 Mar 16;9(3):e25441. doi: 10.2196/25441 (PMC8968549; doi:10.2196/25441)

This is a Multimedia Appendix to a full manuscript published in the JMIR Mental Health. For full copyright and citation information see http://dx.doi.org/10.2196/25441

Home page for patients with core modules “relapse psychoeducation” and “relapse prevention plan”, and the “mood and anxiety diary”

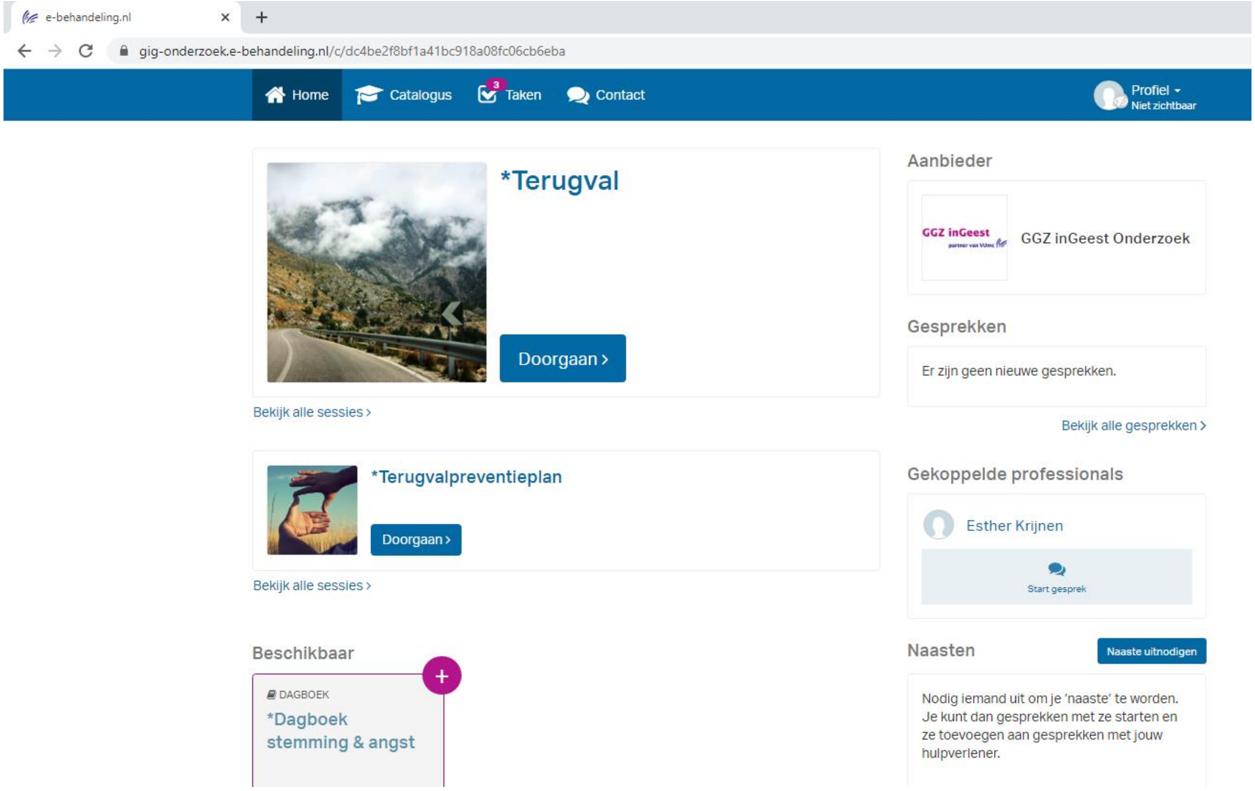

Supplement: Multimedia Appendix 1 [file mental_v9i3e25441_app1.pdf]
