# Supplementary material for: Usage Intensity of a Relapse Prevention Program and Its Relation to Symptom Severity in Remitted Patients With Anxiety and Depression: Pre-Post Study
Source: JMIR Ment Health. 2022 Mar 16;9(3):e25441. doi: 10.2196/25441 (PMC8968549; doi:10.2196/25441)
Supplement: Multimedia Appendix 2 [file mental_v9i3e25441_app2.pdf]

This is a Multimedia Appendix to a full manuscript published in the JMIR Mental Health. For full copyright and citation information see http://dx.doi.org/10.2196/25441

Course of symptoms, also specified by usage intensity

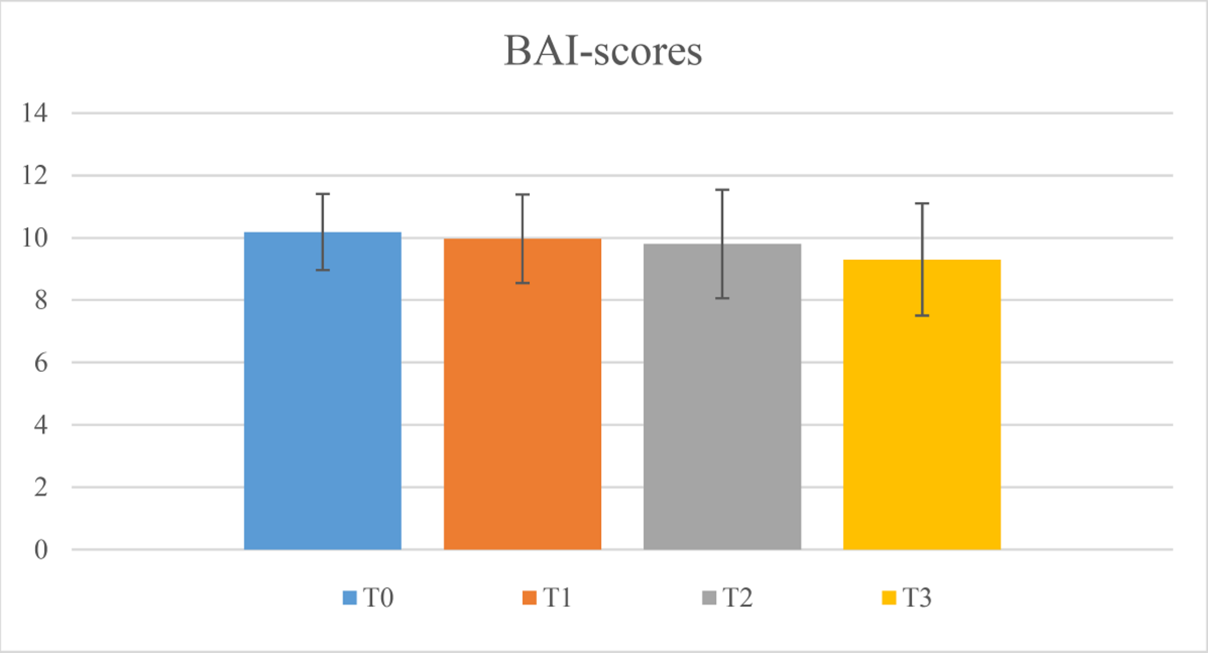

Figure S1: Course of anxiety symptoms over time

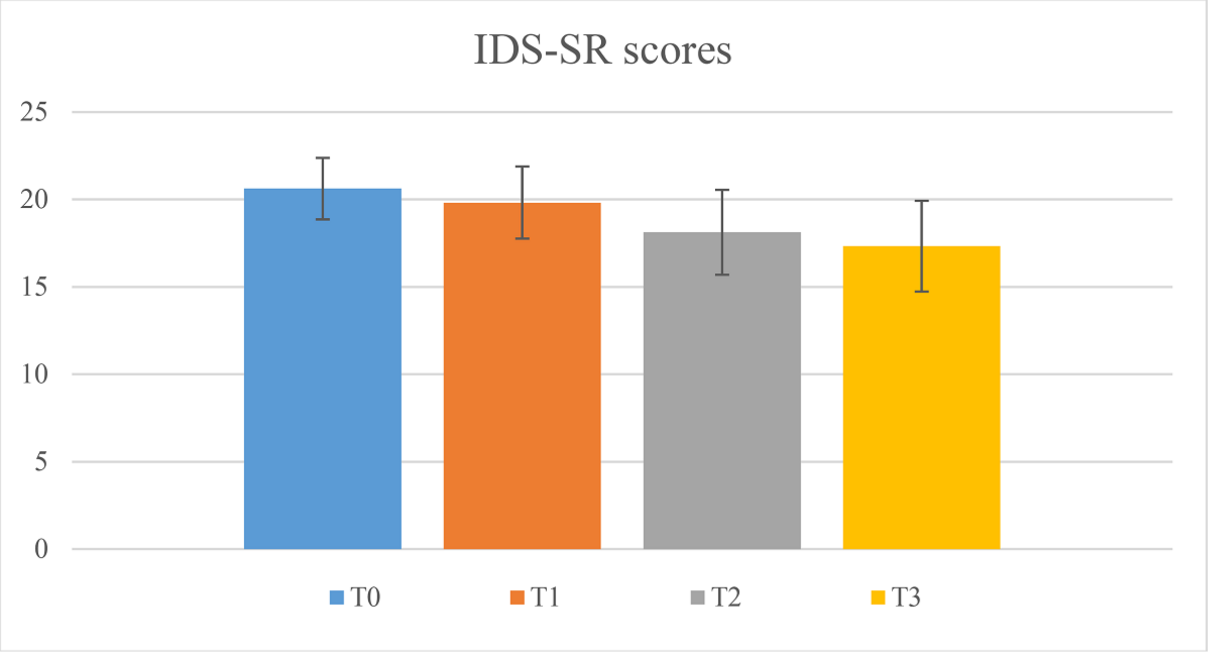

Figure S2: Course of depressive symptoms over time

This is a Multimedia Appendix to a full manuscript published in the JMIR Mental Health. For full copyright and citation information see http://dx.doi.org/10.2196/25441

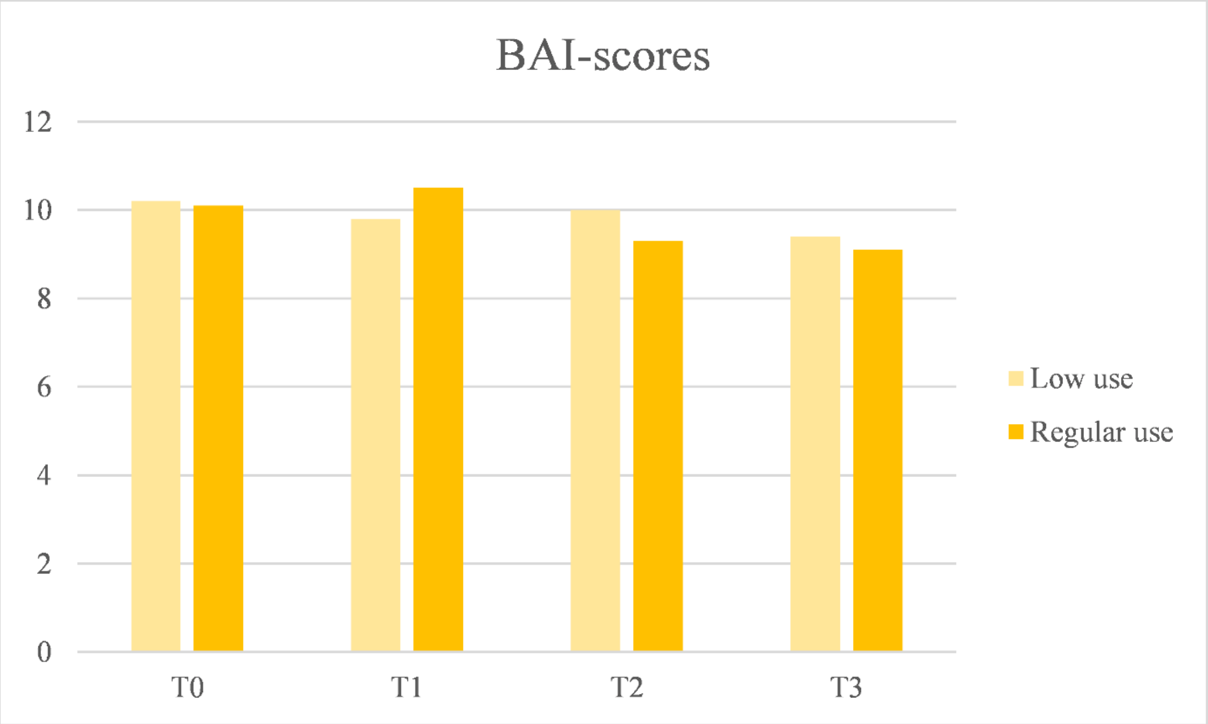

Figure S3: BAI scores for low and regular use according to the minimal usage intensity measure

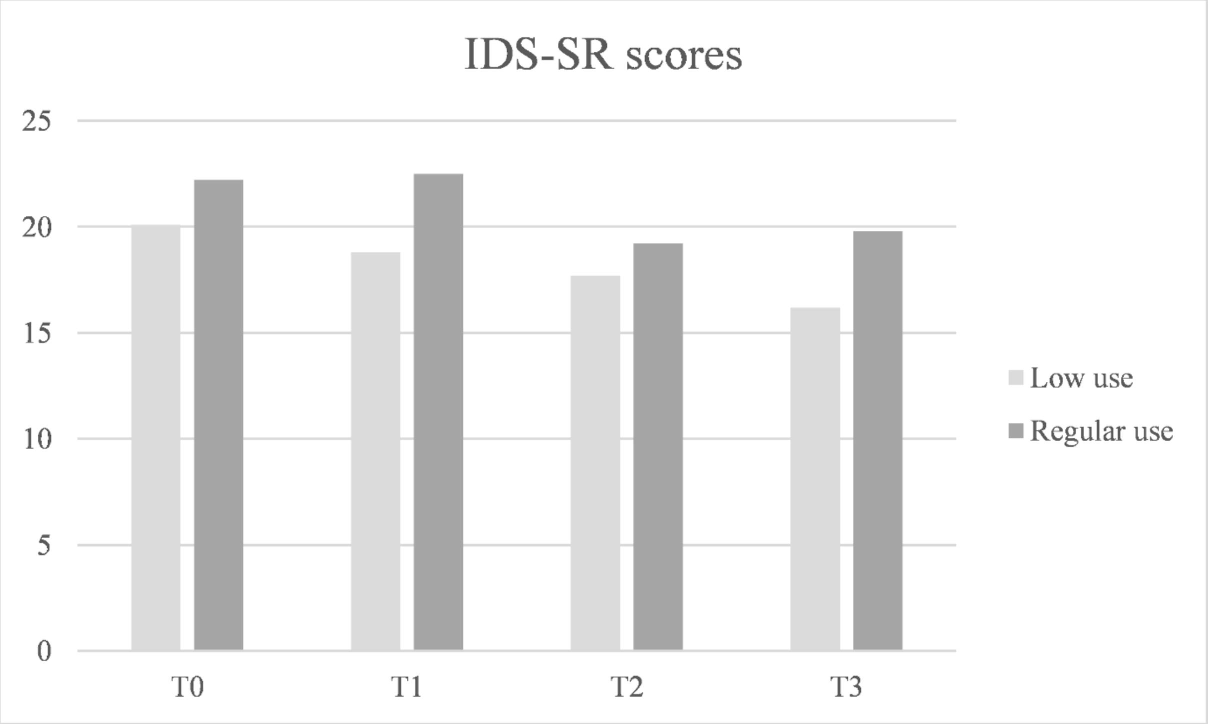

Figure S4: IDS-SR scores for low and regular use according to the minimal usage intensity measure
